# Unlocking the Potential of Macroalgae: Innovative Pretreatment Strategies for Efficient Biorefinery

**DOI:** 10.3390/molecules31050909

**Published:** 2026-03-09

**Authors:** Xiucheng Gu, Ying Zhou

**Affiliations:** Department of Biotechnology, School of Marine Science and Technology, Harbin Institute of Technology, Weihai 264209, China; 23s030073@stu.hit.edu.cn

**Keywords:** macroalgae, biorefinery, pretreatment, bioconversion, fermentation, genetic editing

## Abstract

Macroalgae represent a promising third-generation feedstock for biorefinery due to their high biomass productivity and non-reliance on arable land. However, their complex cell wall structure poses a significant barrier to efficient bioconversion. This review integrates current pretreatment methods, including physical, chemical, biological, and combined approaches, with a focus on their mechanisms, effectiveness, and limitations. Furthermore, it explores the conversion of pretreated macroalgal biomass into bioenergy and biochemicals, such as bioethanol, organic acid and polyhydroxyalkanoate, via microbial fermentation. The review also examines the application of genetic editing tools (e.g., CRISPR-Cas systems) for the targeted modification of macroalgae to improve their inherent characteristics for biorefinery, such as reducing biomass recalcitrance or increasing the content of target carbohydrates. Finally, future perspectives on technological innovations and integrated industrial chains of macroalgal biorefinery are discussed. This review serves as a systematic reference for deepening the understanding of macroalgal cell wall deconstruction processes and supports the development of efficient and environmentally benign pretreatment strategies to advance macroalgal biorefinery toward industrialization.

## 1. Introduction

In recent years, with the exacerbation of the global energy crisis and environmental problems, accelerating the development and use of new types of resources has become a major strategic tool for countries around the world. Biomass, as the only large-scale organic carbon source for biorefineries, possesses the characteristics of being renewable, readily available and abundant [1]. It is, therefore, unsurprising that the focus of scientists in various countries around the world is on effectively obtaining renewable energy and high-value-added compounds through sustainable processing, with a view to making biomass more valuable.

To date, the evolution of biorefining has been primarily categorized into first-generation (utilizing food crops like corn and wheat) and second-generation (utilizing lignocellulosic biomass like agricultural residues) technologies. However, both generations face significant limitations. First-generation biorefineries have raised ethical concerns over “food-versus-fuel” conflicts, posing potential threats to global food security and commodity prices [2]. Although the second-generation biorefinery can relieve the pressure on food resources, it still has some development bottlenecks. First of all, lignocellulosic biomass has a structure that is difficult to decompose, so energy-intensive and costly pretreatment methods must be employed [3]. Secondly, it still takes up arable land. Moreover, the complex composition often adopted leads to form inhibitors during pretreatment, which would reduce the efficiency of microbial fermentation at the subsequent stages [4].

To overcome these challenges, scientists turn attention to the marine sector, and work on the third-generation biorefinery based on algal biomass. Macroalgae are particularly noteworthy due to their promising prospects, compared with the terrestrial biomass, which possess several disruptive advantages. (1) They can grow without arable land or freshwater; (2) they undergo rapid growth, producing good amounts of biomass, and capturing CO_2_ far more efficiently than land plants [5]; (3) their cell walls are unique, containing polysaccharides such as alginate, carrageenan, agar, and generally lacking lignin, making macroalgae theoretically easier to break down [6].

Though having evident potential, the commercialization of macroalgal biorefinery still faces a core problem: inherent recalcitrance caused by their unique cell wall architecture (rich in sulfated polysaccharides, cellulose networks, etc.) and high salinity. These factors together form a natural physicochemical barrier that hinders the bioconversion process [7]. Therefore, efficient and low-cost pretreatment strategies are crucial for breaking down algal structures and releasing fermentable sugars, facilitating value-added utilization.

This review intends to comprehensively summarize the progress in pretreatment technologies for macroalgal biomass and explore its potential for conversion into bioenergy, organic acid and polyhydroxyalkanoate (PHA) via microbial fermentation, with special emphasis on the mechanisms, efficacy and drawbacks of various pretreatment strategies (physical, chemical, biological, and their combinations). Furthermore, prospects such as genetic editing and process intensification are also discussed, with the goal of providing theoretical insights and practical reference for advancing the industrialization of macroalgal biorefinery.

## 2. Chemical Composition and General Structure of Macroalgae

Macroalgae are typically sorted according to their pigmentation: Chlorophyta, Phaeophyta, and Rhodophyta. Overall, Rhodophyta contains the highest number of species, then Chlorophyta and Phaeophyta [2]. Macroalgal chemical composition makeup differs by species. In addition to interspecific differences, different sampling time and location and variations in salinity or cultivation depth cause extensive variations in components.

Table 1 shows the chemical composition of the three types of seaweed. In general, macroalgae have a high moisture content accounting for 50–90% of fresh weight. For dry weight, they are mainly composed of carbohydrates, followed by proteins, minerals, and lipids [8].

**Figure 1:**
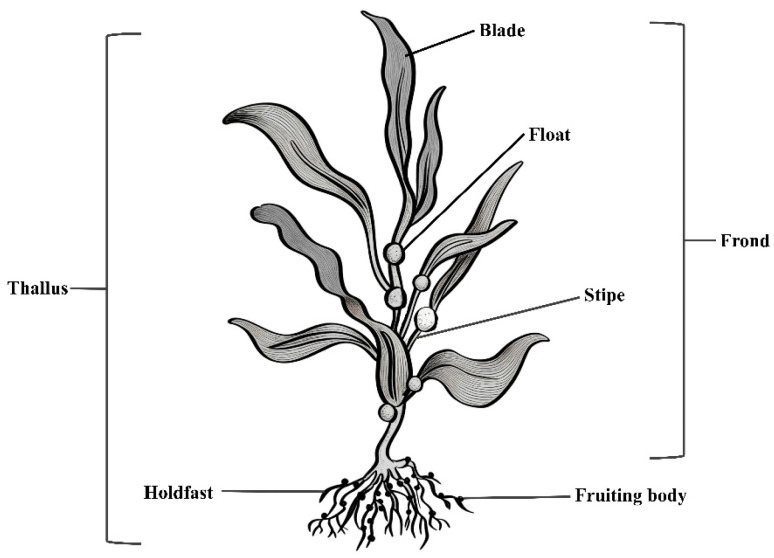
A typical macroalgal structure with its main characteristics (redrawn from [9]).

Figure 1 shows the general structure of macroalgae. With a lack of vascular tissue, macroalgae do not have true roots, stems, and leaves, which means their morphology is characterized by simple reproductive structures instead. In natural environments, they primarily anchor to rocky substrates, although in certain cases they may attach to sandy particles.

**Figure 2:**
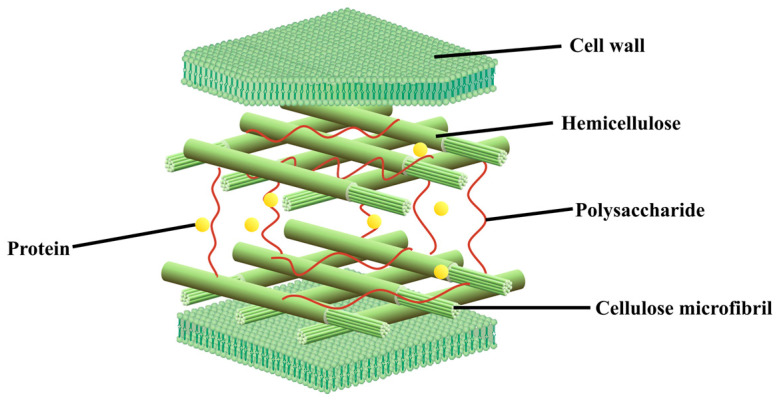
Structure and composition of a typical macroalgal cell (redrawn from [10]).

The structural complexity observed macroscopically originates from the unique biochemical architecture of the macroalgal cell wall. As illustrated in Figure 2, the cell wall is primarily composed of a fibrillar skeleton of cellulose microfibrils and a matrix of polysaccharides and proteins. This intricate network of cellulose microfibrils, amorphous polysaccharides, and structural proteins constitutes the fundamental barrier to efficient bioconversion, necessitating targeted pretreatment strategies to disrupt its integrity and liberate fermentable sugars and other valuable components.

### 2.1. Chlorophyta (Green Seaweeds)

Chlorophyceae, commonly referred to as green seaweeds, represent a major group of macroalgae, boasting as many as 4500 species [7]. Green algae carbohydrate content ranges from 25% to 60% of dry weight, and it mainly consists of polysaccharides such as ulvan and starch. Ulvan consists primarily of α- and β-(1,4)-linked units of rhamnose, xylose, galactose, glucuronic acid and iduronic acid units, along with repeating disaccharides (such as β-D-glucuronic acid (1,4)-linked to α-L-rhamnose 3-sulfate). In contrast, starch and cellulose are mainly composed of glucose units, but with distinct spatial arrangements [8]. In addition to the dominant glucose structure in cellulose, some xylose and mannose units have been found to form linkages via β-1,3 and β-1,4-glycosidic bonds, respectively, acting as structural substitutes for glucose in specific cases [11]. Starch features an open helical structure with weak intermolecular linkages, which renders it more susceptible to degradation through chemical, physical or enzymatic processes [12]. Some of the primary species of Chlorophyceae are *Caulerpa*, *Cladophora*, *Ulva* and *Codium*.

### 2.2. Rhodophyceae (Red Seaweeds)

Rhodophyceae, commonly referred to as red seaweeds, rank as the most abundant and widely distributed group of macroalgae, encompassing approximately 6000 species [13]. These seaweeds are typically grown in deep cold or warm shallow marine environments, often adopting filamentous or sheet-like growth forms and occasionally parasitizing other seaweeds. Beyond their ecological traits, as photosynthetic pigments, they have chlorophyll A and phycobilins. Of their dry weight, 30–70% constitutes carbohydrates, especially polysaccharides such as carrageenan, agar and cellulose. Carrageenan represents a family of linear sulfated polysaccharide consisting primarily of D-galactose (D-Gal) and 3,6-anhydro-D-galactose (D-AHG), with alternating α-1,3 and β-1,4 glycosidic linkages connecting these monomers. Agar is a complex mixture whose gelling fraction is predominantly agarose. Agarose is a structurally simple, linear macromolecular polysaccharide. Its repeating unit consists of D-Gal and 3,6-anhydro-L-galactose (L-AHG), linked by alternating β-1,3 and α-1,4 glycosidic bonds [14]. Some of the primary species of Rhodophyceae are *Gracilaria*, *Pyropia*, *Gelidium* and *Kappahycus*.

### 2.3. Phaeophyceae (Brown Seaweeds)

Phaeophyceae, commonly known as brown seaweeds, represent a group with distinct photosynthetic pigments including chlorophylls (a and c) and carotenoid. There are around 1500 different species [8] and they preferably grow in shallow and cold waters. Phaeophyceae exhibit a highly complex chemical composition with strong seasonal variability. This complexity gives rise to a diversity of minor bioactive compounds, encompassing polar lipids, phenolic compounds, essential minerals and proteins.

Different species of brown macroalgae, which have complex metabolic constitutions, therefore, contain a unique complement of carbohydrates. Alginate, fucoidan and laminarin are the primary polysaccharides found in brown seaweeds [15]. Alginates, the most abundant polysaccharides in Phaeophyceae, consist chiefly of β-D-mannuronic acid (M) and α-L-guluronic acid (G) units linked by 1–4 glycosidic bonds [16]. Fucoidan, a polyanionic sulfated macromolecule with a highly complex chemical structure, comprises primarily fucose and sulfate groups. Its structural characteristics vary significantly across different Phaeophyceae species [17]. Glucose is the main sugar monomer of laminarins joined by (1,6)-linked glycosidic linkages and (1,3)-linked β-glucans. These polysaccharides encompass two chain types: one consisting mainly of glucose, and the other featuring a glucose backbone with D-mannitol residues attached to the reducing terminals [18]. Some of the key species of Phaeophyceae are *Fucus*, *Saccharina*, *Laminaria*, *Himanthalia* and *Sargassum*.

## 3. Drivers of Macroalgal Biorefinery

Biorefinery is characterized as the sustainable processing of biomass into a spectrum of biobased products and bioenergy [19,20]. Biorefinery development encompasses two strategic goals: the first is the replacement of conventional energy sources through the adoption of renewable domestic raw materials (the energy goal), and the second is to establish a robust biobased industry (the economic goal) [21,22]. Thus, the tendency towards a new generation of biorefinery must balance environmental and ecological sustainability through a carbohydrate-centric process that enables the highly efficient conversion of polysaccharides into monomeric sugars (e.g., glucose and xylose) [23,24].

Biorefinery can be divided into four generations, depending on the biomass feedstocks and the processing technology (Table 2). The advantages of macroalgal biorefinery, such as low recalcitrance property, the feasibility of transforming them into renewable energy, the capacity to produce a diverse array of commodities, the ability for carbon dioxide sequestration and the efficiency of resource utilization in cultivation, collectively underpin their indisputable potential [25,26].

Concurrently, national governments have established comprehensive policy frameworks to provide robust support for the advancement of macroalgal biorefinery. Northern Europe has recognized the emerging growth potential of the macroalgae industry and is actively investigating its strategic integration within the blue bioeconomy framework. The Swedish Government has published the Maritime Strategy, which mentions macroalgae and emphasizes its development prospects. The Federal Government of Germany has issued the National Bioeconomy Strategy 2030, which regards algae as a potential raw material source with various applications in medicine, industry, agricultural ecology, and environment, and states that more research is needed to improve the entire algal production chain [27]. In addition, several countries, especially China and Indonesia, have instituted policy frameworks, creating strategic opportunities for the development of macroalgae biorefinery. As the world’s largest seaweed producer, China has brought new opportunities to this field through strategies such as rural revitalization, the construction of a strong maritime nation, Belt and Road Initiative, and other strategies aimed at further advancing macroalgae development [28]. In Indonesia, the world’s second-largest producer, the combination of abundant resources, supportive policies, and technological advancements constitutes the main driving force for developing seaweed biorefinery. The National Energy Policy (NEP No. 79/2014) mentions the goal of increasing the share of new and renewable energy to 23% by 2025 and 2050. The high availability and rapid growth of macroalgae make it the ideal candidate to help achieve this target. This potential is being actively quantified through ongoing research and development, as evidenced by promising biogas yields of 0.100–0.855 m^3^ CH_4_/kgVS and bioethanol production from species like *Gracilaria* and *Ulva* [29].

**Figure 3:**
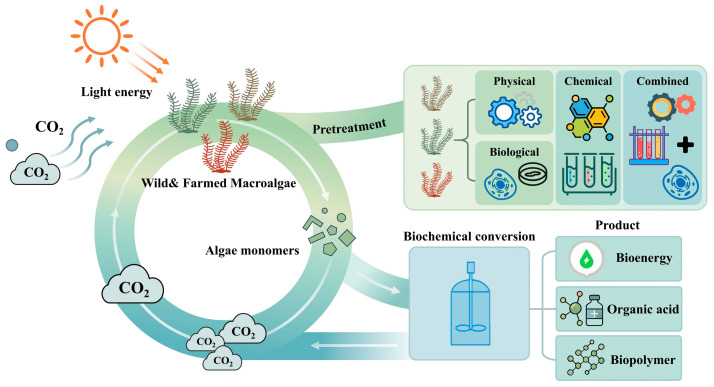
Concept of macroalgal biorefinery.

This robust policy framework acts as a key strategic driver, creating a supportive platform and necessary guarantees for advancing macroalgal biorefinery, thereby enabling the conversion of macroalgae into bioenergy, organic acids, and biopolymers at the current stage (Figure 3) [30].

## 4. Challenges of Macroalgal Biorefinery

**Figure 4:**
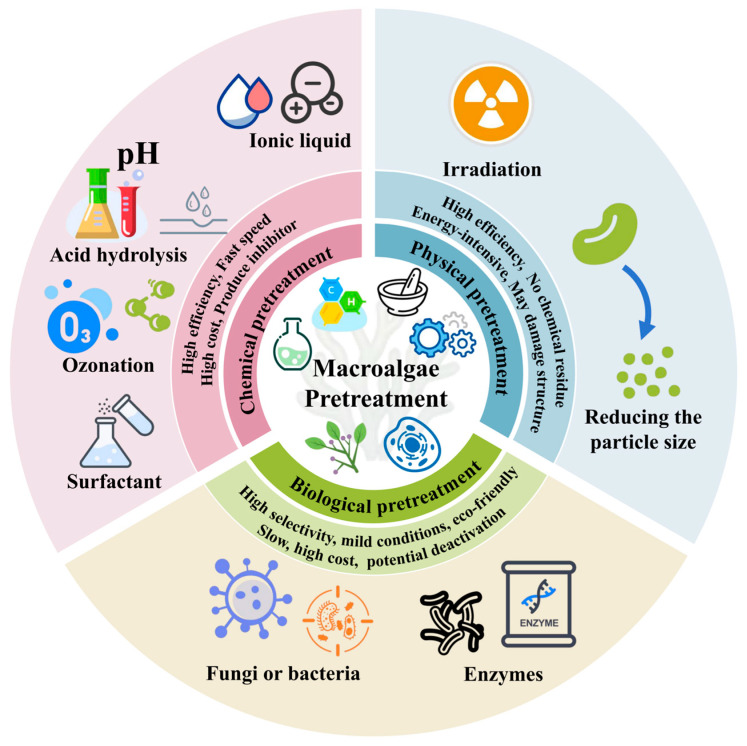
Advantages and limitations of various macroalgae pretreatment techniques.

Pretreatment is an crucial upstream operation in the macroalgal biorefinery, which is used to break down the cell wall structure (Figure 4). The main purpose is to increase the bioavailability of organic matter by breaking down complex structural polysaccharides in order to facilitate subsequent microbial fermentation. However, the efficiency of pretreatment is often affected by some inherent challenges particular to macroalgal biomass [31].

A major challenge is the structural diversity and complexity of macroalgal cell walls [32]. These cell walls account for at least 50% of the dry weight and serve as an important carbon reserve, which are mainly composed of polysaccharides [33]. Some polysaccharides are found only in macroalgae [34]. Green algae cell walls comprise matrix-sulfated xylo-arabinogalactans alongside a diverse array of polysaccharides such as sulfated galactans, sulfated glucans, sulfated arabinan, and sulfated arabinogalactans [35]. Red algae cell walls are predominantly composed of the sulfated galactans agars [36,37,38]. Also, the hemicellulose and sulfated glucans are also components related to Rhodophyta cell walls [5]. Brown algae cell walls are primarily made up of alginate and fucoidans [37,39]. The significant heterogeneity in chemistry and structure poses a fundamental challenge to the development of pretreatment technologies. It means that pretreatment process optimized for one type of macroalgae (e.g., brown algae) may be entirely ineffective when applied to another (e.g., red or green algae). This species specificity necessitates the development of “customized” pretreatment methods for different macroalgae, even the same species from different locations, greatly increasing the time and funding needed for process development. Furthermore, depending on the desired target product (such as an efficient saccharification process for biofuel production), the choice of pretreatment conditions also becomes more complex, further complicating the establishment of a standardized technological framework. As a result, this impedes the industrialization of macroalgae biorefinery.

Another issue comes from intrinsic inhibitor compounds in macroalgae, such as phenols, minerals, sulphates, and halogenated compounds. These compounds can be released during pretreatment, thereby inhibiting downstream fermentation processes [40,41,42]. High salinity, as a component of marine biomass, has also attracted attention, as it can affect both the kinetics of pretreatment reactions and the microbial activity in subsequent stages [43]. Importantly, the pretreatment can lead to the formation of undesirable inhibitory compounds (e.g., furfurals from degradation of sugar), which are harmful to fermentation.

Therefore, addressing the intertwined challenges of biomass heterogeneity, inherent and process-induced inhibition, and economic viability is of paramount importance. The development of robust, efficient, and cost-effective pretreatment strategies therefore stands as a critical and indispensable prerequisite for unlocking the full potential of macroalgal biorefinery.

## 5. Recent Advances in Pretreatment Techniques

### 5.1. Physical Pretreatment

Macroalgae can be processed with physical pretreatment, which involves the application of external forces to disrupt the structure of the seaweed and reduce its crystallinity and polymerization. This process has been shown to increase subsequent conversion, with the advantages of continuous processing, high solids loading, the absence of fermentation inhibitors, rapid processing, the non-requirement of additives, and a small, non-expanding, water-soluble fraction of the treated powders, which improves hydrolysis efficiency. Common physical pretreatment techniques include milling, extrusion, and irradiation [44].

Although the physical pretreatment methods generally comprise simple steps and cause fewer environmental problems, such methods consume substantial amounts of energy. Therefore, understanding macroalgal physical properties and technique is important for the rational selection of physical pretreatment for the chosen macroalgae. Such a selection serves to ensure a sound balance between cost efficiency and process performance.

#### 5.1.1. Reducing the Particle Size

The efficacy of mechanical size reduction for macroalgae depends upon the macroalgal species. Researchers have found that the application of the same milling technique to different macroalgae species might have varying effects and might even have a negative effect on sugar extraction [45]. Research has shown that the beneficial value of mechanical size reduction is governed by the cell wall structure, as cell walls with higher fiber content have a greater impact on mechanical pretreatment in sugar production [46]. Milling of *Laminaria digitata* (large flat blade Phaeophyceae) had no effect on its surface area or glucose release, as the wet refiner milling merely caused two-dimensional scission of the blades without significantly increasing the overall surface area for enzyme attack. This could be attributed to the blade thickness being smaller than the milling disc distances used, limiting three-dimensional defibrillation [47]. The varied outcomes are even more evident in comparative studies: while beating (a wet mechanical pretreatment) improved methane yield for *Laminaria* spp., ball milling (which involves prior drying) reduced methane production by 21–27%, likely due to overly rapid hydrolysis and acidogenesis which inhibited methanogenesis. In contrast, maceration significantly increased methane yield for *Ulva lactuca* (green seaweed) [48], and physical pretreatment like washing and maceration enhanced methane production for *Gracilaria vermiculophylla* (red seaweed) by increasing surface area [49]. These different reaction mechanisms can be attributed to differences in cell wall structure. Brown algae such as *Laminaria* contain alginate as a major matrix polysaccharide, which may require specific enzymes (e.g., alginate lyase) for effective degradation rather than mere particle size reduction. Additionally, high ash and mineral content in macroalgae can affect the behavior of particles after pretreatment, such as agglomeration via ionic bonding, thereby impacting the accessibility of substrate. Therefore, the effect of mechanical pretreatment depends on algal morphology, pretreatment methods, and target conversion processes, necessitating tailored approaches for each macroalgae.

Amamou et al. compared the effects of two mechanical pretreatment methods (vibro-ball and centrifugal milling) on the ethanol yield of two macroalgae species, and concluded that for *Gelidium sesquipedale*, centrifugal milling hydrolyzed it more efficiently than vibro-ball milling, increasing total sugar and glucose release by up to 129% and 261%, respectively. In contrast, neither pretreatment method showed any significant effect on *U. lactuca* [50]. The cell walls of red algae are rich in complex polysaccharides such as carrageenan and agar. Intensive mechanical forces can effectively disrupt this dense network structure, significantly increasing the accessible surface area for enzymatic action. Conversely, the intrinsic high ash content of green algae induces a mineral–vegetal co-milling effect during grinding, meaning that the untreated raw material itself is already sufficiently accessible to enzymatic action, thereby diminishing the incremental benefits of additional pretreatment.

Rodriguez et al. demonstrated that novel mechanical pretreatment using a Hollander beater significantly enhanced the anaerobic digestion of the brown macroalgae *Pelvetia canaliculata* [51]. The study noted that methane yield from macroalgae typically ranges between 120 and 480 mL/gVS. Under the optimized conditions (50 min at a feedstock-to-inoculum ratio of 0.3), a maximum methane yield of 283 mL/gVS was achieved, representing a 45% increase over the untreated control (196 mL/gVS). This optimized yield corresponds to approximately 59% of the upper end of the cited typical theoretical range.

#### 5.1.2. Irradiation

Among all the reported physical pretreatment methods, irradiation is viewed as an attractive choice for macroalgal pretreatment [52]. Irradiation pretreatment is more accessible and efficient due to its high energy efficiency, ease of operation and high selectivity. In this method, high-energy radiation is used to break the chemical bonds of macroalgae. The effects of irradiation mainly depend on parameters such as radiation frequency, exposure duration and macroalgal composition. There are three methods of irradiation: (1) microwave, (2) sonication, and (3) gamma rays.

Microwave pretreatment shows considerable potential for macroalgae, because they have high water content, which allows for rapid heating and pressurization, which can aid in the disruption of the cell wall [53]. Yin et al. studied the impact of microwave pretreatment (100–180 °C for 30 min) on the disintegration of *L. japonica*. They found that pretreatment significantly disrupted the algal cell walls and promoted the release of soluble organic matter. Especially for xylose and arabinose, which are important fermentable sugars, they increased significantly at temperatures of 140 °C and above. As a result of the improvement in substrate accessibility and the provision of readily biodegradable sugars, there was a substantial rise in microbial dehydrogenase activity. Consequently, the largest hydrogen yield was 15.8 mL/g total solids added (TS_added_) at 160 °C, which was 1.9 times more than the untreated control. This demonstrates that microwave pretreatment effectively enhances biohydrogen production by improving the saccharification and bioavailability of macroalgal biomass [54]. However, the hydrogen yield achieved was only about 4.5% of its theoretical maximum. This huge gap reveals a broader reality: although microwave pretreatment effectively doubled the actual hydrogen output by improving saccharification and bioavailability, the overall conversion efficiency of the single-stage dark fermentation process remains low. As noted in the study, more than 90% of the energy either remains in the algal biomass or is converted into soluble metabolites rather than hydrogen. This highlights the fact that, although pretreatment is crucial for the initial disruption of the biomass structure, pretreatment alone cannot fully unlock the energy potential of the substrate. To achieve theoretical hydrogen yields, integrated biorefinery strategies are essential for maximizing energy recovery from macroalgae. Moreover, the efficacy of pretreatment was evidenced in the study on *Eucheuma denticulatum* residues, where microwave-assisted pretreatment improved the enzymatic saccharification efficiency. The key metric of enzymatic digestibility was elevated to 96.5% under optimized pretreatment, resulting in a glucose yield that was twice as high as that from the untreated control. This direct enhancement of sugar release, achieved without the formation of inhibitory byproducts, was the fundamental reason behind the efficient downstream fermentation [55].

Ultrasonic pretreatment has high energy and strong penetration, through the thermal effect, cavitation effect and mechanical effect produced to destroy the chemical bond of seaweed and reduce the degree of crystallinity, and its effect is related to the type of seaweed, ultrasonic frequency and the treatment time. Short pretreatment time, mild operating conditions, green environment, and effective decomposition of seaweeds are the advantages of ultrasonic pretreatment, but there is a problem of high cost for large-scale reaction. Paletta et al. demonstrated that the efficacy of ultrasonic pretreatments on *Sargassum* spp. varied significantly. Sonication pretreatment resulted in a 5.28% increase in methane yield, an improvement attributed to acoustic cavitation that mechanically disrupts cell walls and enhances biodegradability. This positive effect starkly contrasted with microwave pretreatment, which reduced yield by 26.06% due to the formation of recalcitrant compounds [56]. Likewise, expanding on the theme of ultrasonic pretreatment, an investigation by Zaidi et al. on *Enteromorpha* concluded that a significant increase in the solubilization of organic compounds (a critical precursor for subsequent microbial conversion) was achieved under optimized conditions (5 min, 30% amplitude, 20:1 liquid–solid ratio), resulting in a maximum cumulative biogas production of 373 mL with a biohydrogen content of 32.7% (*v*/*v*). However, prolonged sonication (15 min) has been shown to be detrimental. Excessive mechanical and thermal energy input led to the degradation of solubilized organic matter into inhibitory byproducts, which can acidify the digester system and directly suppress methanogenic archaea, while soluble phenolic compounds are known to be toxic to anaerobic microorganisms. This shift in the metabolic pathway ultimately curtailed the final energy yield, underscoring the necessity for precise optimization of pretreatment intensity [52].

There have been limited reports on gamma ray irradiation. The gamma radiation pretreatment uses γ-photons to produce the Compton, photoelectric and electron-pair effect with the seaweed, destroying the compact structure of the seaweed. The process is straightforward, without the involvement of chemicals, has little impact on the environment and produces no inhibitors, but the effect of single treatment is limited; it is better to use it in combination with other pretreatment methods. Moreover, the economic viability of this pretreatment needs to be enhanced for large-scale industrial application. Gamma irradiation at 10–30 kGy was found to be effective in destroying the structure of macroalgae, thus providing more usable organic compounds for hydrogen production. However, higher doses of irradiation (30 kGy) are actually unfavorable for hydrogen production. In conclusion, gamma irradiation can help to disrupt the structure of macroalgae and increase hydrogen production in fermentation, but it is crucial to optimize the irradiation dose based on a comprehensive evaluation of substrate disintegration and the response of the microbial community [57].

### 5.2. Chemical Pretreatment

Among all pretreatment methods, chemical pretreatment is the most widely studied technique. Compared to other methods, researchers are continuously exploring more efficient and compatible compounds for degrading macroalgae.

#### 5.2.1. Acid Hydrolysis

Macroalgae are usually pretreated with inorganic acids (sulfuric, hydrochloric, nitric or phosphoric) at concentrations of 0.1–5.0% *w*/*v* in combination with high temperatures [58,59,60,61,62,63,64]. However, a significant drawback of this method is the concomitant generation of fermentation inhibitors, such as formic acid, furfural, HMF (5-hydroxymethylfurfural), etc., which can hinder subsequent microbial processes.

In exploring this challenge, Kooren et al. compared various acid pretreatments across different seaweeds [65]. They found that the optimal acid is highly species-dependent; sulfuric acid was the best chemical for *U. fasciata* and *G. corticata*, whereas orthophosphoric acid was most effective for *S. wightii*.

Building on the need for optimized and sustainable pretreatment solutions, Greetham et al. developed a novel seawater-based acid pretreatment that significantly enhances saccharification efficiency in macroalgae. Using 5% sulfuric acid in seawater at 121 °C for 15 min, hydrolysis yields reached 64.63% for *L. digitata*, 69.19% for *U. linza*, and 63.03% for *Porphyra umbilicalis*, representing a 12–23% increase compared to reverse-osmosis water controls. Furthermore, in a demonstration of practical application, fed-batch fermentation of concentrated *U. linza* hydrolysate with the marine yeast *Wickerhamomyces anomalus* M15 achieved a record ethanol titer of 48.24 g/L (yield: 0.329 g/g sugar) [61]. This approach not only improves efficiency but also highlights a dual benefit by reducing freshwater dependency and enabling high-productivity marine biorefining.

Due to the strong corrosiveness of inorganic acid pretreatment, recycling difficulties and other shortcomings, in recent years, researchers have increased the study of organic acids and solid acids. Organic acids, which can be green-synthesized, are relatively friendly to the environment, generate less toxic substances and are better matched with the subsequent enzymatic activity. Among them, methanesulfonic acid (MSA) has been used many times in macroalgae pretreatment [66,67]. Meinita et al. investigated the production of levulinic acid (LA_1_) and formic acid (FA) from *Kappaphycus alvarezii* using MSA pretreatment. Through optimization, they achieved maximum yields of 14.69% for LA_1_ and 5.35% for FA under the conditions of 180 °C, 0.6 M MSA, 30 min, and 2.5% biomass loading. This high efficiency is attributed to MSA’s strong acidity and ability to promote the saccharification process [66]. Similarly, in a study on the red macroalga *G. verrucosa*, Park et al. demonstrated even higher efficiency using MSA. Under optimized conditions (180 °C, 0.5 M MSA, 20 min, 10% biomass), they achieved a remarkable levulinic acid yield of 22.02% (based on biomass), further underscoring the exceptional catalytic performance of MSA in macroalgal biorefinery [67].

Although the process of biomass degradation using inorganic acids is relatively mature, its industrial application is constrained by equipment corrosion and environmental pollution. Therefore, solid acid pretreatment, as an environmentally friendly process, has attracted widespread attention due to its advantages of high selectivity, stability, and reusability. The development of solid acid catalysts, such as zeolite, ion-exchange resin, solid superacid, metal oxide, heteropolyacid, and carbon-based solid acid, has, thus, become a key research focus [68].

Solid acids such as Amberlyst [69,70,71], Dowex (TM) Dr-G8 [72], HZSM-5 [73,74], and MCM-41 [75] have been used for the pretreatment of macroalgae, and significant results have been seen. Park et al. focused on the production of LA_1_ using hydrothermal conversion with Purolite CT269DR as the catalyst. By optimizing the conversion conditions, a 30.3% (22.58 g/L) yield of LA_1_ was obtained at 200 °C for 90 min with 12.5% biomass and 50% catalyst loading of biomass quantity. At the same time, FA yielded 14.0% (10.42 g/L) [76]. The effectiveness of solid acid catalysts is highly dependent on the choice of reaction medium. In the conversion of alginic acid, Kim et al. introduced an innovative γ-butyrolactone (GBL)/water co-solvent system, which synergized with the Amberlyst-15 solid acid catalyst to significantly enhance furfural production efficiency [69]. They found that in a GBL/H_2_O (1:1 *v*/*v*) system, the yield of furfural reached 32.2%, much higher than in pure aqueous or other solvents. The researchers pointed out that adding GBL can effectively suppress the undesirable side reactions of furfural degradation, thereby improving the reaction selectivity. The results of this study provide critical insights into refining biomass conversion through the use of eco-friendly solvents.

#### 5.2.2. Ozonation

Ozone pretreatment is an emerging approach that can improve the biomass through oxidation, which effectively solubilizes and degrades recalcitrant cell walls. Unlike acid hydrolysis, it would not produce any fermentation inhibitors. Instead, ozonolysis primarily generates weak carboxylic acids, causing limited damage to sugar polymers. Although ozone is highly reactive, the process lacks substrate selectivity, meaning it attacks various components of the biomass matrix non-specifically. Sulfahri et al. reported that after ozone pretreatment (1% substrate loading, 400 mg O_3_/L, 0.5 L/min, 60 min, 30 °C) of *Kappaphycus alvarezii* and *Gelidium amansii*, the sugar yield increased by 3.2-fold and 6.8-fold, respectively, compared to untreated biomass, showcasing its efficacy in improving saccharification [77].

#### 5.2.3. Ionic Liquid

Ionic liquids (ILs) have been widely recognized as eco-friendly solvents in recent years, owing to their unique properties, including low melting points, wide liquid range, non-volatility, and high stability [78]. IL pretreatment primarily refers to the disruption of complex algal polysaccharide structures by anions with strong polarity and high hydrogen-bond basicity [79]. Although IL pretreatment has the advantages of high efficiency and minimal generation of inhibitory compounds, it also faces many obstacles such as high cost, complex synthesis, and the toxicity of some ILs. Therefore, reducing the cost of ILs, achieving efficient recovery, and developing low-toxicity ILs are urgent priorities for the development and application of ILs [80].

Weldemhret et al. combined IL pretreatment and enzymatic hydrolysis for selective sugar production from *Gelidium amansii*. Using [Bmim]Ac at 90 °C for 12 h can achieve 99% biomass dissolution. Subsequent hydrolysis with an optimized recombinant agarase cocktail (33.3% Aga2, 33.3% AgaA7, 33.3% Aga50D; total 50 U/g) followed by α-neoagarobiose hydrolase AhgI (2.5 U/g) yielded 56.5% D-galactose and 33.7% 3,6-anhydro-L-galactose—significantly higher than conventional methods. The IL was successfully recycled five times, and the efficiency was still over 80% after pretreatment, which verified the sustainability of the process [81]. While ILs show broad prospects in pretreatment, improving the processing efficiency remains a core challenge. In this regard, Uju et al. proposed an excellent solution [82]. They found that for carrageenan industry seaweed waste, direct enzymatic hydrolysis achieved 77% conversion rate. However, by introducing a preactivation step with 1.9% (*v*/*v*) peracetic acid (PAA) at 80 °C for 3 h, followed by treatment with 1-hexylpyridinium chloride ([Hpy][Cl]) at 100 °C for 30 min, the conversion rate was boosted to 91%. This strategy enhances the pretreatment efficacy of ILs through PAA-mediated activation, providing a clear direction for designing high-efficiency and low-consumption ILs pretreatment processes suited to specific feedstocks.

#### 5.2.4. Surfactant

Adding surfactants can reduce the surface tension as well as the interfacial tension between the molecules, in turn expanding the available surface area. At present, research mainly focuses on the combined application of surfactants and mechanical pretreatment to macroalgae [83,84].

Gondi et al. investigated the potential use of *U. fasciata* for biohydrogen production and its decomposition under ultrasonic disintegration (UD) and ultrasonic combined surfactant disintegration (UCSD) using alkyl polyglycoside (APG) [85]. Their optimized UCSD conditions employed 10 μL of APG surfactant combined with 160 W ultrasonic power for 30 min. This combined approach achieved a maximum soluble chemical oxygen demand (SCOD) release of 1030 mg/L and a biomass liquefaction of 21.2%, significantly higher than UD alone. They concluded that UCSD (121.6 mL) produced far more biohydrogen than UD (90.4 mL) and the control group (17.3 mL). This enhancement was achieved by the surfactant effectively disrupting the rigid cell wall structure, which augmented the ultrasonic effect and released more readily fermentable organic substrates. Similarly, Pugazhendi et al. applied Pluronic P-123-induced microwave pretreatment to decompose *U. reticulata* [86]. Their optimized conditions employed a Pluronic P-123 dosage of 0.06 g/g TS combined with a microwave power of 0.36 kW for 10 min. It was found that the addition of this surfactant greatly increased the COD solubilization by approximately 10% (from 22.33% to 31.02%) and significantly reduced the required reaction time, obtaining an optimal hydrogen yield of 98.37 mL.

### 5.3. Biological Pretreatment

Biological pretreatment employs microorganisms (fungi or bacteria) and/or enzymes to selectively degrade specific components of the algal biomass. These processes are highly efficient, consume low energy, are eco-friendly, and can be conducted under either aerobic or anaerobic conditions. The choice of enzymes depends on the primary constituents of the seaweeds, such as cellulose, xylan, glycoproteins, and fucoidan.

Several studies have explored the application of biological pretreatment in macroalgae [87,88,89]. Sulfahri et al. concluded that fungal pretreatment by *Trichoderma harzianum*—which secretes a suite of enzymes including cellulase, xylanase, and β-glucosidase—followed by enzymatic hydrolysis of *K. alvarezii* and *G. amansii* produced sugar contents of 0.55 g/g and 0.53 g/g, respectively. These enzymes act in synergy to break down the structural polysaccharides of the algal cell wall into fermentable monosaccharides. Consequently, fungal pretreatment prior to enzymatic hydrolysis boosted sugar yields by up to 2.3 times in comparison to enzymatic hydrolysis without fungal pretreatment [87]. Teune et al. recently proposed a novel streamlined process [90]. They utilized a specific endo-1,4-β-xylanase to hydrolyze *Palmaria palmata*, and this process efficiently converted mixed-linkage xylans into xylooligosaccharides (XOSs) with a degree of polymerization of 2–4. The process achieved a notable XOS yield of 17.6% (176 mg/g biomass). Moreover, by adding a β-xylosidase that prefers to degrade 1,4-linked components, it increased the yield to 22.6% and enriched the final product with 1,3-linked and mixed-linkage XOS for specialized applications.

### 5.4. Combined Pretreatment

Single pretreatment has its limitations, such as high cost, environmental pollution, toxicity, or poor effectiveness. These drawbacks hinder its application in large-scale production. To address these problems, dual or multiple pretreatment technologies, known as combined pretreatment, has gained increasing attention in recent years.

A combined mechanical–chemical pretreatment was reported on *U. reticulata* which utilized a disperser along with dilute sulfuric acid to produce biohydrogen [31]. Mechanical pretreatment was performed at 12,000 rpm for 30 min. Based on this, the chemical pretreatment was conducted by adjusting the biomass slurry to pH 5 with acid. This synergistic strategy yielded biohydrogen of 60 mL/gCOD. However, *U. reticulata* pretreated with the disperser alone produced 30 mL/gCOD and untreated produced 5 mL/gCOD, far lower than the samples with the combined method. In an experiment combining ultrasound and oxalic acid pretreatment (OSP) to produce methane from *G. salicornia*, Gondi et al. found this combination to be highly effective [91]. Specifically, ultrasound pretreatment (SP) was optimized under the conditions of 140 W for 30 min, and then combined with 0.06 N oxalic acid. This OSP strategy brought SCOD to 914 mg/L (16.93% solubilization), a substantial increase in the release of carbohydrates (205 mg/L) and proteins (118 mg/L), and yielded the highest methane production of 0.252 L/gCOD. This final methane yield was 81% higher compared to SP alone (0.139 L/gCOD) as well as over ten times higher than the unpretreated control group (0.022 L/gCOD).

In order to systematically evaluate the pros and cons of pretreatment approaches, Table 3 provides a comparative analysis based on key indicators such as sugar yield and inhibitor formation.

Though combined pretreatment methods can enhance the availability of fermentable substrates, the optimal strategy for selecting pretreatment techniques should prioritize processes that minimize water usage, reduce chemical input, lower energy demand, and have lower capital/operating cost.

The paradigm shift in the field of macroalgal biorefinery pretreatment is no longer limited to proposing new methods solely within physical, chemical, or biological pathways. Instead, it emphasizes innovative combinations of these methods. The emerging trend leverages the structural disruption capability of physical pretreatment, the targeted polysaccharide depolymerization of chemical pretreatment, and the eco-friendly specificity of biological pretreatment within a system framework. Its main goal is to develop customized and hybrid pretreatment systems that not only enhance processing efficiency and yield but also significantly reduce energy consumption, chemical usage, and environmental impact, thereby paving the way for economically viable and sustainable biorefinery.

The development of pretreatment technologies for lignocellulosic biomass has been rapid, and these technologies also provide valuable references for the processing of macroalgae [92]. Techniques such as ammonia fiber expansion (AFEX) [93] and steam-CO_2_ explosion [94], though still in the early stages of application to macroalgae, show great potential for technology transfer, as the methods have been proven to effectively break down complex biological structures and offer new pathways for the efficient utilization of macroalgae.

## 6. Genetic Editing Tools for Macroalgal Biorefinery

**Figure 5:**
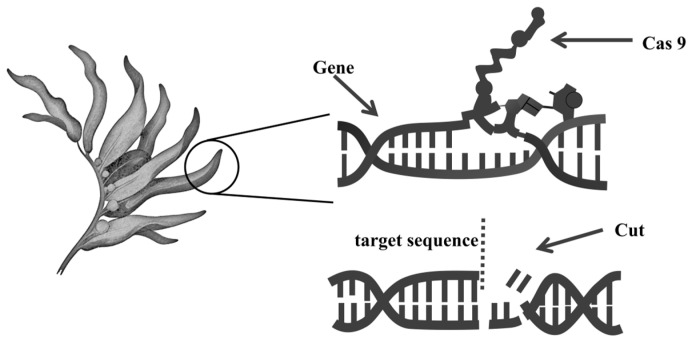
Schematic diagram of macroalgal genetic editing technology.

While optimizing the pretreatment process remains crucial, genetic editing offers a transformative approach for the efficient biorefinery of macroalgae by altering cell wall structure and enhancing carbohydrate accessibility, thereby enabling its fundamental and targeted modification (Figure 5).

The advancement of genetic editing tools has provided new avenues for precise genetic alteration of macroalgae [95]. The CRISPR-Cas system stands as the most promising technology because it is highly specific, effective, and easier to design. A stable transformation and CRISPR-based knock-in system was successfully established in the green algae *U. prolifera*, where a bacterial antibiotic-resistance gene (*aph7*”) was integrated in the genome with an endogenous promoter in order to generate knock-in mutants resistant to the co-selection of the antibiotic hygromycin B and 2-fluoroadenine [96]. In addition to green algae, platforms for gene editing are also being set up in red macroalgae. In *Gracilariopsis lemaneiformis*, the CRISPR/LbCas12a system was applied to edit the carbonic anhydrase (*ca*) and phycoerythrin (*pe*) genes. By delivering preassembled ribonucleoprotein (RNP) complexes via microparticle bombardment, Zhang et al. achieved targeted mutations, including base substitutions, insertions, and deletions. Notably, when editing the *pe* gene, which encodes a pigment protein, there are phenotypic changes that can be observed under fluorescence microscopy, facilitating the screening of edited mutants [97]. Similarly, foundational breakthroughs have been made in brown algae. Badis et al. established the first CRISPR-Cas9-based gene editing platform in the model brown alga [98]. They achieved targeted gene knockouts by directly delivering preassembled Cas9 RNP complexes via biolistics or microinjection. A key innovation was the development of an efficient dual-mutation strategy: they first targeted the *ADENINE PHOSPHORIBOSYL TRANSFERASE* gene, utilizing 2-fluoroadenine resistance conferred by its mutation for positive selection. This system was then successfully employed to isolate double mutants harboring concurrent mutations in the *APT* locus and a second, non-selected target gene (e.g., *FKBP12*, *VBPO*, or *MAS1*).

In summary, the CRISPR-Cas system has made great achievements in macroalgae, and it has moved from the proof-of-concept stage to the initial application stage, and is in the dawn of a new genetic editing era for seaweeds. These studies generally employ strategies that directly deliver preassembled RNP complexes to bypass the hurdle of not-yet-fully established stable genetic transformation systems. Furthermore, positive selection systems constructed from endogenous genes also provide an important tool for effective screening of edited individuals. But there are both basic and technical problems in the field. Fundamental challenges include inherent characteristics of macroalgae such as their long growth cycles and immature genetic transformation systems. Technical challenges include species-specific differences in delivery efficiency, the lack of efficient homology-directed repair systems, and insufficient understanding of endogenous regulatory elements.

It should be noted that, at present, the focus of research is on the establishment of methods and editing reporter genes. No modification has been made to the genes related to carbohydrate metabolism pathways. However, in related organisms, such as microalgae, studies have successfully changed the carbon flux by knocking out genes for either storage polysaccharide synthases or degradation enzymes, using CRISPR with great success in increasing the concentration of desired carbohydrates or lipids [99,100,101]. And this provides a very strong technical basis for macroalgae: through precise editing, it can fundamentally edit the structure of the algal cell wall and change the carbohydrate composition in order to achieve the goal of strategically designing raw materials to reduce pretreatment difficulty and increase fermentable sugar content.

In the future, improving delivery methods, creating species-specific CRISPR tools, and developing effective platforms for the simultaneous editing of multiple genes and base editing will be important directions for overcoming current obstacles and achieving accurate breeding. On this basis, it is recommended to conduct in-depth analyses of the synthesis and regulation mechanisms of cell wall polysaccharides in macroalgae, find key metabolic pathway targets, and finally achieve targeted improvements of algal raw materials via genetic editing, such as knocking out synthases of hard-to-degrade polysaccharides or inhibiting their degradation pathways, creating new routes for building a sustainable biorefinery system from high-quality raw materials to efficient conversion, thereby finally achieving the grand vision of efficient biomass biorefinery.

## 7. Macroalgae Biorefinery Products

### 7.1. Bioenergy

The excessive reliance on non-renewable fossil fuels is exacerbating economic and environmental risks, so there is an urgency to shift to renewable and cleaner sources to alleviate the pressure [91,102]. Macroalgae, characterized by rapid growth rates and unique capacity for photosynthetic CO_2_ sequestration, serve as ideal feedstocks for the production of bioenergy, such as ethanol and biohydrogen, via microbial fermentation, providing an efficient pathway to sustainable energy. Table 4 shows the pretreatment and fermentation methods for bioenergy production from macroalgae.

Bioethanol has received much attention for its environmental benefits. After dehydration, macroalgae are pretreated and fermented, then distilled to ethanol [64,116]. The production of ethanol from macroalgae is an effective way to address sustainability issues, as the preparation of bioethanol from macroalgae has been shown to release less greenhouse gas than traditional sources [117]. However, achieving economic competitiveness remains a core bottleneck in the country’s industrialization process. A techno-economic analysis focusing on the brown macroalgae *Nizimuddinia zanardini* revealed a stark economic contrast between two process pathways [118]. Simulation results indicated that a standalone “only-fuel” scenario (producing solely fuel ethanol and electricity) is economically unviable, with a maximum allowable dry seaweed price (MDSP) of $64 per ton. This suggests that, even with free feedstock, the process cannot be profitable, mainly due to the low conversion rate of seaweed carbohydrates to ethanol, which is insufficient to offset the high processing and capital costs through a single product. In contrast, adopting an “integrated biorefinery” scenario dramatically improves economics. This model allows the MDSP to rise significantly to $374 per ton (under a target internal rate of return of 20%).

Due to the minimal or very low lignin content of macroalgae, macroalgae have a great advantage in the production of biogas. The biogas generated by algae is primarily composed of methane, which contains the most significantly decreased carbon as well as contributes about 65%, and CO_2_, which represents the highest oxidized and fluctuates by about 35% [102,119]. Meanwhile, the positive effects associated with biogas production from algal fermentation also include the potential use of the residual biomass after anaerobic digestion to enhance soil properties. The digestate contains large amounts of elements such as nitrogen, phosphorus, and potassium, which are beneficial for plant growth. It can help to improve soil fertility and reduce the reliance on synthetic fertilizers, thereby supporting a circular bioeconomy [120]. Shivaranjani et al. adopted a combined pretreatment of low temperature (80 °C) and mechanical pretreatment (disperser at 10,000 rpm) to produce biogas from *Codium*. Within 15 min, the release of SCOD reached 1360 mg/L, achieving a 17% liquefaction rate. Consequently, the pretreated biomass showed a higher biogas yield, reaching 238 mL/gCOD, substantially greater than that of the untreated sample (28 mL/gCOD) and the sample subjected to low-temperature pretreatment alone (182 mL/gCOD) [115].

Biobutanol, a biologically synthesized fuel, remarkably mimics petrol and holds numerous potential applications [121]. Marine macroalgae have the potential to be converted into butanol through acetone–butanol–ethanol (ABE) fermentation. ABE fermentation is a two-phase process that produces butyric and acetic acids during the acidogenic process.

During the initial acidogenic phase, anaerobic bacteria produce volatile fatty acids (VFAs), primarily acetic acid and butyric acid. Subsequently, in the solventogenic phase, these acids are metabolically converted into ABE. Khaonuan et al. conducted a comparative assessment of butanol production from three Thai marine macroalgae (*G. tenuistipitata*, *U. intestinalis*, and *Rhizoclonium* sp.) using *Clostridium beijerinckii* ATCC10132. They found that *G. tenuistipitataas* achieved an initial butanol titer of 1.38 g/L under pretreatment conditions (80 °C for 5 min). More significantly, through optimization, they determined that hydrothermal pretreatment at 110 °C for 10 min with a solid-to-liquid (S/L) ratio of 120 g/L could achieve the maximum reducing sugar conversion rate of 57.6%. This optimal pretreatment ultimately led to a substantial increase in butanol production of 3.1 g/L [107]. A study by Jiang et al. achieved record-high butanol production from red seaweed hydrolysate [108]. The hydrolysate was prepared through a process of acid pretreatment (2% H_2_SO_4_, 121 °C, 30 min) of *G. lemaneiformis*, followed by concentration and detoxification with activated carbon. Subsequent fermentation using the specialized strain *Clostridium* sp. NJ4 yielded 12.56 g/L of butanol and 19.86 g/L of total ABE solvents.

Biohydrogen, as a clean and renewable energy source with near-zero emissions from hydrogen fuel cells and as an intermediate energy transport vehicle for preserving and transmitting renewable energy sources, is increasingly being recognized worldwide [122]. Margareta et al. obtained the highest hydrolysis efficiency with a reducing sugar yield of 0.21 g RS/g of biomass by pretreating the *Ulva* sp. with 4% H_2_SO_4_ and 121 °C for 40 min, and the highest cumulative hydrogen yield (2340 mL/L) was obtained by fermentation with *C. butyricum* CGS5 [123].

### 7.2. Organic Acid

Macroalgal biomass can be converted through microbial fermentation into high-value organic acids, including lactic acid, pyruvic acid, and succinic acid. These organic acids serve as key synthetic precursors in the food and pharmaceutical industries, demonstrating considerable commercial application value [124,125].

Table 5 summarizes recent reports in the production of organic acids from macroalgae. As shown in Table 5, various macroalgae species have been converted into high-value organic acids through different pretreatment methods followed by microbial fermentation using specific strains (e.g., *Lactobacillus rhamnosus*, *Weissella paramesenteroides*, *Bacillus coagulans*). Notably, *L. rhamnosus* achieved a lactic acid (LA_2_) yield of 0.85 g/g from *Ulva* sp. hydrolysate, while *W. paramesenteroides* reached 0.94 g/g using *Gracilaria* sp. These values significantly surpass the conversion efficiency of lignocellulosic feedstocks (0.18–0.19 g/g), offering innovative solutions for sustainable and economically viable LA_2_ production [126]. Additionally, other organic acids, such as succinic acid, pyruvic acid, and citramalic, also showed considerable production potential. These results suggest that macroalgae have broad application prospects as sustainable raw materials for the production of organic acids in biorefinery.

Although macroalgae have potential in organic acid production, their application still faces key challenges, including low pretreatment efficiency and the tendency to produce inhibitors, as well as the lack of robust industrial strains capable of efficiently utilizing specific sugars. Improvements lie in developing green pretreatment technologies to minimize inhibitor formation as much as possible, and in using synthetic biology to construct high-performance cell factories that can broadly utilize various substrates and withstand complex environments.

### 7.3. Biopolymers

**Figure 6:**
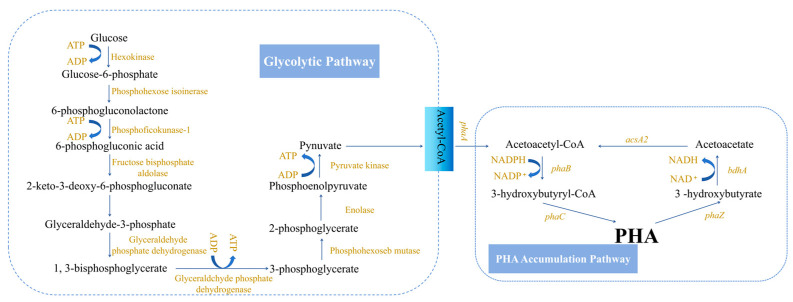
The metabolic pathway for PHA fermentation.

The metabolic pathway of PHA fermentation from sugars is illustrated in Figure 6. PHA is a natural polymer biomaterial produced in the process of microbial metabolism, stored in the cytoplasm in the form of particles, with good biocompatibility, biodegradability and mechanical properties, so that it can be used in a wide range of applications, not only as a biomedical material, but also as a biodegradable packaging material, and has become one of the most active hotspots of research in the field of biomaterials in recent years [133,134,135].

The study by Jeong et al. presented a highly promising technical approach for PHA production from macroalgae [136]. They successfully constructed an alginolytic enzyme complex comprising two different alginate lyases (AlyA1 and AlyA5) and immobilized it on the cell surface of *Ralstonia eutropha*, thereby enabling consolidated bioprocessing for the efficient production of PHA from brown algae *Ecklonia cava*. This immobilized enzyme complex exhibited excellent thermal stability (maintaining more than 60% of its activity after 6 h at 60 °C) and effectively converted alginate into the main intermediate 4-deoxy-L-erythro-5-hexoseulose acid (DEH). Ultimately, the engineered strain utilized alginate as the sole carbon source to achieve a PHA yield of 2.58 g/L. This research points out the potential of using enzyme engineering and strain modification to turn alginate-rich Phaeophyceae into PHA.

Table 6 presents the diverse pretreatment approaches and promising yields achieved in PHA production from macroalgae, highlighting the potential of marine biomass as a sustainable raw material for biopolymer production. Although the potential for PHA production from macroalgae is considerable, its commercialization fundamentally depends on economic feasibility. The traditional bacterial fermentation processes for PHA is costly, with organic carbon sources and mineral salts accounting for approximately 50% of the total production costs [137,138,139].

In comparison, macroalgae as a raw material source have significant economic and environmental advantages, and can serve as low-cost or even zero-cost raw mate-rials (e.g., using aquaculture waste or invasive species). However, when scaling up production, downstream processing remains the major cost bottleneck. The harvesting and drying steps can contribute 20–30% and about 20% to the overall biomass produc-tion price, respectively [137]. Furthermore, PHA extraction often involves enormous quantities of toxic and volatile solvents, which increases total production costs and raises environmental concerns. To overcome these hurdles, adopting an integrated bi-orefinery model is considered crucial for achieving economic feasibility.

Macroalgae have enormous potential to be an eco-friendly feedstock for producing high-value products, including bioenergy, organic acid, and biopolymers (PHA). However, there are still many challenges on the path to commercialization, even though numerous successes have been reported in the laboratory. Traditional acid pretreatment often produces inhibitors that impact downstream fermentation and raise environmental issues. While enzymatic hydrolysis offers an eco-friendly alternative, its cost remains high due to the expense of industrial enzymes (e.g., cellulases and alginate lyases). Furthermore, progress in developing recombinant microbial strains that can efficiently utilize carbohydrates from diverse macroalgae lags behind that for terrestrial biomass. A primary barrier to commercialization is insufficient validation of scalability. Most studies are limited to small-scale operations, which cannot address critical questions related to process reliability, yield, and economic viability. In addition, there may be potential conflicts when extracting other components while targeting biorefinery products, which requires careful allocation of resources.

In the future, the success of biorefinery will depend on collaboration across different disciplines, integrating microbiology, engineering, and economics. Key strategies include the following: (1) Strain selection: obtaining native microbial strains isolated from marine environment (such as seawater and algal surfaces), where complex macroalgal sugar metabolism naturally occurs; (2) Co-fermentation: using microbial consortia or engineered strains to jointly metabolize glucose and specific monosaccharides from macroalgae, thereby degrading polysaccharides and achieving optimal product yield.

## 8. Future Perspectives

### 8.1. Technological Breakthrough

Achieving technological breakthroughs in macroalgal biorefinery relies on developing conversion processes that are highly efficient, economical, and environmentally friendly. However, the diversity of potential products is inherently closely related to the specificity of the pretreatment methods used. Therefore, future research must focus on the in-depth optimization and fundamental innovation of pretreatment technologies to substantially reduce the production costs and capital investment for algal-based bioenergy and biochemicals. Key directions include exploring novel green pretreatment solvents, engineering robust microbial cell factories capable of utilizing diverse macroalgal sugars, and employing artificial intelligence for integrated control and optimization of pretreatment and fermentation processes.

Furthermore, to bridge the gap from the laboratory to industry, addressing scale-up engineering challenges is important. For the chemical and thermochemical pretreatment methods discussed, this notably includes the following: (1) developing corrosion-resistant materials and reactor designs to withstand reagent environments; (2) managing the rheological properties of macroalgal biomass slurries (e.g., highly viscous suspensions) to achieve effective mixing, heat transfer, and mass transfer in large-scale operations; (3) integrating energy-efficient strategies for methods with high energy input (e.g., certain physical or thermochemical processes); and (4) designing robust processes for the recovery and recycling of chemicals (e.g., ionic liquids) to improve economic viability and environmental sustainability. Future research should pay attention to conducting pilot-scale demonstration work to effectively overcome these operational-level barriers, thereby bridging the gap between promising laboratory-scale results and commercially viable biorefining processes.

### 8.2. Industrial Chains

Currently, the seaweed processing industry generates a large amount of waste each year, which requires effective treatment to reduce environmental pollution and resource waste. Numerous macroalgae residues also contain high-value components, including carbohydrates, proteins, minerals, and lipids. Such constituents hold potential application prospects as nutritional supplements, fertilizers, animal feed, and raw materials for industrial production [147]. Therefore, in the future, the entire seaweed component can be utilized through an industrial chain.

In addition, the production of bioenergy and biochemicals from seaweed is only economically viable if the harvesting costs of algae biomass are low. Biomass harvesting is determined by whether the macroalgae are free-drifting or surface-attached. Land-based and nearshore macroalgal cultivation practices typically capture coastal areas utilized for aquaculture, so offshore cultivation practices are the preferred approach for macroalgal cultivation. Offshore cultivation is further regarded as cost-effective, owing to its straightforward installation process and low maintenance costs. In addition to offshore cultivation, co-cultivation of diverse macroalgal species has also increased biomass yields by enhancing light collection efficiency [148].

## 9. Conclusions

An examination of different generations of biorefineries reveals that third-generation biorefinery, represented by macroalgae, demonstrates significant advantages in terms of environmental sustainability and resource cost-effectiveness. A review of existing research and demonstration projects indicates that the main research focus in this field remains the determination of the optimal pretreatment and fermentation process conditions, aiming to achieve efficient and high-value conversion of algal resources at the lowest cost.

This review focuses on the macroalgal pretreatment, genetic editing and conversion into bioenergy and biochemicals via microbial fermentation, with an emphasis that while macroalgal cell walls generally lack lignin, their unique structure still imparts inherent recalcitrance, which requires efficient pretreatment methods. Furthermore, it is noteworthy that the carbohydrate composition of macroalgal hydrolysates is complex (e.g., glucose, galactose, fucose), while microorganisms usually exhibit substrate preference and prioritize easily degradable monosaccharides like glucose. Therefore, to advance the sustainable development of macroalgal biorefinery, future research should focus on the following: first, the process optimization and targeted modification of abundant macroalgal species; second, the development of efficient, environmentally friendly, and economically viable pretreatment technologies; third, the fermentation of hydrolysates using tailored microorganisms to promote the complete component utilization of macroalgae; and, fourth, the implementation of comprehensive lifecycle assessments and techno-economic evaluations to enhance the sustainability and economic feasibility of the macroalgal biorefining process.

## Figures and Tables

**Table 1:** The chemical composition of the three types of macroalgae [7,8,9].

Type	Green Seaweed	Red Seaweed	Brown Seaweed
Water content	60–80%	60–90%	50–75%
Carbohydrate content	25–60% dry weight	30–70% dry weight	30–76% dry weight
Proteins	25–50% dry weight	10–47% dry weight	3–15% dry weight
Ash content	18.0–49.6%	0.7–28.2%	8.7–41.2%
Lipids	0.1–6.7%	0.3–7.4%	0.1–7.9%
Photosynthetic Pigments	Chlorophyll A Chlorophyll B Carotenoids	Chlorophyll A Phycobilins	Chlorophyll A Chlorophyll C Carotenoids (mainly fucoxanthin)
Main Polysaccharides	Ulvan Starch	Carrageenan Agar	Alginate Fucoidan Laminarin
Important Species	*Halimeda* *Ulva* *Codium* *Caulerpa*	*Gracilaria* *Palmaria* *Gelidium* *Kappahycus*	*Fucus* *Saccharina* *Laminaria* *Himanthalia* *Sargassum*

**Table 2:** Evaluation of biorefinery generations across sustainability and technological dimensions.

Generation	Sources	Advantages	Disadvantages	Technological Maturity	Environmental Sustainability	Industrialization Prospects
First generation	Food crops	Mature technology; easy conversion.	Competition with food production; high environmental cost.	High	Low	Mature but limited
Second generation	Lignocellulosic biomass	No direct competition with food; utilize waste.	Complex pretreatment and conversion technology; significant challenges in feedstock collection and logistics.	Moderate	Medium to high	Medium to high
Third generation	Algae biomass	No land required; high efficiency in CO_2_ absorption.	Technology in early research and development stage; energy-intensive processes of cultivation, harvesting, and processing.	Moderate	High	High
Fourth generation	Genetically optimized algal biomass	Aimed for “carbon negative” emissions.	Potential biosafety concerns; technology at the cutting-edge stage.	Low	(Theoretical) high	High

**Table 3:** Comparison of the effect of pretreatment methods on macroalgae.

Macroalgae	Pretreatment Methods	Sugar Yield	Inhibitory Compounds	Ref.
*G. sesquipedale*	Vibro-ball milling	13.59 g/g TS		[50]
*E. denticulatum residues*	Microwave-assisted autohydrolysis	Glucose 19.30 g/L		[55]
*L. digitata* *U. linza* *P. umbilicalis*	Dilute acid pretreatment	20.81 g/L 26.23 g/L 24.02 g/L		[61]
*K. alvarezii* *G. amansii*	Ozone pretreatment	0.52 g/g dry algae 0.41 g/g dry algae		[77]
*K. alvarezii* *G. amansii*	Acid pretreatment	0.56 g/g dry algae 0.51 g/g dry algae	0.0488 g/g 5-HMF 0.0511 g/g 5-HMF	[77]
*G. amansii*	Ionic liquid pretreatment	D-Galactose 56.5% 33.7% 3,6-Anhydro-L-galactose		[81]
*K. alvarezii* *G. amansii*	Fungal pretreatment	0.55 g/g 0.53 g/g		[87]

**Table 4:** Valorization of macroalgal biomass into bioenergy.

Product	Macroalgae	Pretreatment Method	Microorganism Used	Yield	Ref.
Ethanol	*G. elegans*	Acid hydrolysis (10% H_2_SO_4_, 140 °C, 60 min)	*Saccharomyces cerevisiae*	14.13 g/L	[103]
Ethanol	*Ecklonia maxima*	Steam extraction (118 °C, 1 bar, 10 min)	*S. cerevisiae*	7.3 g/L	[104]
Ethanol	*L. digitata*	Enzymatic treatment	*Thermoanaerobacterium* AK17 _M6	22.5 g/L	[105]
Ethanol	*S. wightii*	Alkali hydrolysis (80 ℃, 70.65 min)	*Lachnoclostridium phytofermentans* KSM 1203	13.75 g/L	[106]
Butanol	*G. tenuistipitata*	Hydrothermal pretreatment (110 °C, 10 min)	*Clostridium beijerinckii* ATCC 10132	3.1 g/L	[107]
Butanol	*G. lemaneiformis*	Acid hydrolysis (2% H_2_SO_4_, 121 °C, 30 min)	*Clostridium* sp. NJ4	12.56 g/L	[108]
Butanol	*G. amansii*	Acid hydrolysis (3% butyric acid, 130 °C, 30 min)	*Clostridium* sp. WK	4.48 g/L	[64]
2,3-butanediol	*L. japonica*	Acid hydrolysis (1% H_2_SO_4_, 120 °C, 18 min)	*Vibrio alginolyticus* X511	14.83 g/L	[109]
2,3-butanediol	*G. amansii*	Acid hydrolysis (180 mM H_2_SO_4_, 150 °C, 10 min)	*S. cerevisiae* BD4	14.80 g/L	[110]
Biohydrogen	*L. japonica*	Gamma irradiation (20 kGy)	Anaerobic sludge	14.17 mL/g-TS_added_	[57]
Biohydrogen	*K. alvarezii*	Hydrothermal gasification (360 °C); followed by photocatalytic reforming	Microbial sludge	61.25 wt%	[111]
Biohydrogen	*U. reticulata*	Mild acid dispersion pretreatment	Methanogenic bacterium	60 mL/gCOD	[31]
Biomethane	*E. maxima*	Enzymatic treatment	Anaerobic microbial community from cow manure	862 mL CH_4_/gVS	[112]
Biomethane	*G. manilaensis* *Gracilariopsis persica*	Acid hydrolysis (5 M HCl, 100 °C, 1 h)	Anaerobic fermentative bacteria	0.281 Nm^3^/kgVS 0.237 Nm^3^/kgVS	[113]
Biomethane	*G. salicornia*	Oxalic acid coupled sonication pretreatment	Anaerobic fermentative bacteria	0.252 L/gCOD	[91]
Biogas	Macroalgae waste	Cutting mill	Anaerobic digested sludge	227 mL/gVS	[114]
Biogas	*Codium* sp.	Low-temperature coupled mechanical pretreatment	Anaerobic bacterium	238 mL/gCOD	[115]

**Table 5:** Valorization of macroalgae biomass into organic acids.

Product	Macroalgae	Pretreatment Method	Microorganism Used	Yield	Ref.
Lactic acid	*Ulva* sp. *Gracilaria* sp. *S. cristaefolium*	Acid hydrolysis (4% H_2_SO_4_, 120 °C, 20 min)	*L*. *rhamnosus* *W*. *paramesenteroides* *L. plantarum*	0.85 g/g 0.94 g/g 0.81 g/g	[126]
Lactic acid	*U. fasciata* *G. corticata* *K. alvarezii*	Acid hydrolysis (1% H_2_SO_4_, 120 °C, 15 min)	*L. plantarum* MTCC 1407	0.38–0.40 g/g 0.51–0.66 g/g 0.61–0.66 g/g	[127]
Lactic acid	*E. denticulatum*	Microwave-assisted thermal pretreatment (120 °C, 50 min) followed by enzymatic hydrolysis	*B*. *coagulans* ATCC 7050	14 g/L	[55]
Succinic acid	*Chaetomorpha* sp.	Microwave pretreatment (microwave dose 1.45 W/g)	ragi tapai consortium	0.65 g/L	[128]
Succinic acid	*P. palmata*	Acid hydrolysis (0.1 N HCl, 121 °C, 15 min)	*Escherichia coli* KLPPP|ΔldhA, ΔpflB, Δpta-ackA, ΔpoxB, pck+ (overexpressed)	22.40 g/L	[129]
Succinic acid	*L. japonica*	Acid hydrolysis (0.1 N HCl, 121 °C, 15 min)	*E. coli* BS002|ΔldhA, ΔpflB	17.44 g/L	[130]
Pyruvic acid	*U. reticulata*	Acid hydrolysis (0.5 M H_2_SO_4_, 120 °C, 90 min)	*Halomonas* sp. BL6	55.23 g/L	[131]
Citramalate	*S. japonica*	Grinding	*Vibrio* sp. dhg VXHC|CimA3.7+ (overexpressed), ΔldhA, ΔfrdABCD, ΔackA-pta, ΔleuCD	0.47 g/g	[132]

**Table 6:** Valorization of macroalgae biomass into PHA.

Macroalgae	Pretreatment Method	Microorganism Used	PHA	Ref.
*Ulva* sp.	Acid hydrolysis (2% H_2_SO_4_, 121 °C, 30 min)	*Cobetia* isolate no.105	0.17 g/L	[140]
*Ulva* sp.	Subcritical hydrolysis (170 °C, 20 min)	*H. mediterranei* ATCC 33500	0.104 g/g	[141]
*Ulva* sp.	Acid hydrolysis (5% H_2_SO_4_, 170 °C, 20 min)	*H. mediterranei* ATCC 33500	0.107 g/g	[142]
*E. spinosum*	Enzymatic hydrolysis	*Halomonas* sp. YLGW01	3.88 g/L	[143]
*Rugulopteryx okamurae*	Enzymatic hydrolysis	*Cupriavidus necator* CECT 4635	0.288 g/L	[144]
*Sargassum* sp.	Acid hydrolysis (5% H_2_SO_4_, 121 °C, 20 min)	*B*. *pacificus* NAA2 *Klebsiella quasipneumoniae* NAA4	72.7% 70.7%	[145]
*E. spinosum* waste	Acid hydrolysis (3M HCl, 121 °C, 15 min)	*C*. *necator* CECT 4635	0.26 g/L	[146]

## Data Availability

No new data were created or analyzed in this study. Data sharing is not applicable.
